# Identification and Validation of Prognostic Markers for Endometriosis-Associated Ovarian Cancer

**DOI:** 10.7150/ijms.97024

**Published:** 2024-07-22

**Authors:** Huilin Yang, Yue Deng, Ying Dong, Yiqun Ma, Lihua Yang

**Affiliations:** Department of Gynecology, the Second Affiliated Hospital of Kunming Medical University, Kunming, Yunnan, 650000, China.

**Keywords:** Endometriosis, Endometriosis-Associated Ovarian Cancer, Prognosis, ADAMTS19, TUBB

## Abstract

**Background:** Growing evidence suggests that endometriosis (EMs) is a risk factor for endometriosis-associated ovarian cancer (EAOC). The aim was to identify and validate gene signatures associated with EMs that may serve as potential biomarkers for evaluating the prognosis of patients with EAOC.

**Methods:** The data of EMs and control samples was obtained from GEO database. The weighted gene co-expression network analysis (WGCNA) identified modular genes significantly associated with EMs. The KEGG pathway and GO functional enrichment analyses were also performed. Univariate Cox regression analysis was conducted to screen marker genes associated with the prognosis of EAOC patients. Finally, RT-qPCR and immunohistochemical verified the expression of ADAMTS19 and TUBB in normal ovarian and EAOC tissues, and the biological functions of ADAMTS19 and TUBB were preliminarily explored by CCK8 and Transwell assays.

**Results:** The WGCNA identified 2 co-expression modules, which in total included 615 genes, and 7642 differentially expressed genes (DEGs) were detected thorough analysis of the EAOC dataset. After taking the intersection of 615 modular genes and 7642 DEGs, 214 shared genes were obtained, and univariate COX regression analysis pointed 10 genes associated with the prognosis of EAOC. Moreover, it was demonstrated by RT-qPCR and immunohistochemical staining experiments that ADAMTS19 expression was elevated, while TUBB expression was reduced in EAOC compared with normal ovarian cells and tissues. Finally, cell experiments revealed that ADAMTS19 promoted the proliferation and invasion in EAOC cells, while overexpression of TUBB inhibited these processes.

**Conclusions:** The present study identified and validated new EMs-associated gene markers, which could serve as potential biomarkers for assessing the prognostic risk of EAOC patients. In addition, some of these genes may have significance as novel therapeutic targets and could be used to guide clinical applications.

## Introduction

Endometriosis (EMs) and ovarian cancer are two significant diseases in gynecology. EMs is chronic and refractory, which shares features with cancer, including metastasis-like behavior, tissue invasion, proliferation, decreased angiogenesis and apoptosis^1^. Large epidemiological studies have indicated that women with EMs have an increased risk of developing epithelial ovarian cancer^1^-^4^. This epithelial ovarian cancer, which is histologically closely related to and may be malignantly transformed from EMs, has been uniformly referred to as endometriosis-associated ovarian cancer (EAOC), with clear-cell carcinoma and endometrioid carcinoma of the ovary being the predominant pathologic types^5^, ^6^. Studies have shown that women diagnosed with clear cell carcinoma are usually younger at an earlier stage than those diagnosed with high-grade serous carcinoma (HGSOC). Early-stage patients with EAOC generally have a favorable prognosis, however, the mortality rate significantly increases when diagnosed at an advanced stage (stage III or IV), with clear cell carcinoma associated with a mortality rate even twice that of HGSOC^7^, ^8^. Therefore, early detection and diagnosis are crucial for the treatment of patients with EAOC.

Numerous previous studies have reported that EMs is associated with 15%-50% of EAOC, and patients with EMs are 2-3 times more likely to develop EAOC^9^-^12^. In Murakami *et al.*'s study, 75% of patients with endometriotic cysts progressed to EAOC within 5 years, and most patients progressed within 10 years^13^. This data suggests that some susceptibility factors in EMs may trigger the occurrence and development of EAOC. Mandai *et al.* reported that microenvironmental factors in ovarian endometriotic cysts play an important role in the malignant transformation of EMs to EAOC, and that the accumulation of aged blood in the cysts facilitates higher concentration of free iron, which leads to sustained oxidative stress, thus converting EMs to EAOC^14^. There was also evidence of immune cell dysfunction in women with EMs, with an imbalance of T-cells leading to abnormal secretion of cytokines (TNF-α, IL-8, VEGF, etc.), causing inflammation, and inflammatory factors are able to promote the growth and progression of EAOC^15^. Kajiyama *et al.* reported that molecular events such as mutations in PTEN, K-ras, and HNF-1β are involved in the malignant transformation of EMs to EAOC^16^.

**Figure 1:**
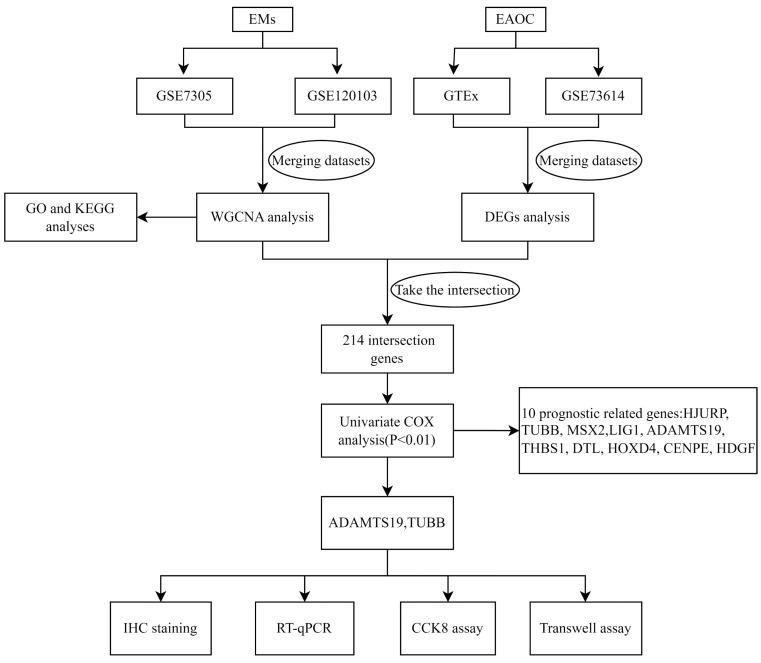
Study flowchart.

Although clinical and epidemiological evidence of a link between EMs and ovarian cancer, the lack of highly sensitive and specific biomarkers to detect ovarian cancer that develops from malignant transformation in patients with EMs. In this study, we identified pivotal genes associated with the prognosis and biological functions in the malignant phenotype of EAOC by bioinformatics methods and cellular experiments. Figure 1 shows the flowchart for the research. The findings of this study may be instructive in considering new biomarkers to predict the prognosis and identify therapeutic targets in patients with EAOC.

## Materials and Methods

### Data acquisition and preprocessing

The datasets were obtained from the National Center for Biotechnology Information (NCBI) Gene Expression Omnibus (GEO) database. We searched with the keywords “ovarian endometriosis” and “ovarian without endometriosis”, and datasets with a sample size greater than 10 and experiment type of Expression profiling by array were included in the study, and those that did not meet the criteria were excluded. These included EMs datasets GSE7305, consisting of 20 samples (10 patients and 10 normal samples), and GSE120103 comprising 36 samples (18 patients and 18 normal samples). For EAOC, We searched with the keywords “ovarian clear cell carcinoma” and “ovarian endometrioid carcinoma”, and excluded datasets that lacked patient survival time and survival status. As a result, we utilized dataset GSE73614 for EAOC, which comprised a total of 103 samples, consisting of 37 clear cell carcinomas and 66 endometrioid carcinomas. Gene expression profiling data for normal ovarian tissues were downloaded from the Genotype-Tissue Expression (GTEx) database, including 88 samples. Table 1 summarizes the detailed information of the four datasets. The data were processed using R software (version 4.3.1) for annotation, analysis, filtering, background correction, log2 transformation and normalization. In addition, datasets GSE7305 and GSE120103, GSE73614 and GTEx were merged separately, and batch effects were eliminated from the merged data utilizing the ComBat method within the "sva" package.

### Weighted gene co-expression network analysis of EMs

WGCNA (Weighted gene co-expression network analysis) is able to cluster genes and construct modules based on similar gene expression patterns and analyze the association between modules and specific traits or phenotypes^17^. In this study, the R package "WGCNA" was utilized to construct the gene co-expression network of EMs. First, the standard R function "Hculst" was used for hierarchical clustering to evaluate whether there were obvious outliers. Second, in order to make the gene expression relationship conform to the scale-free network, the "PickSoftThreshold" function was used to select the appropriate soft thresholding power β. Third, the gene expression similarity matrix based on the soft threshold parameter β is converted to an adjacency matrix using the "adjacency" function. The adjacency matrix obtained in the previous step was converted into a topological overlap matrix (TOM) to minimize the effects of noise and spurious associations. Finally, the hierarchical clustering and dynamic tree cut function detection modules were used to analyze the correlation between the Pearson correlation analysis module and the clinical characteristics of the patients using (*P* <0.05).

### GO and KEGG enrichment analysis of EM-associated genes

Gene Ontology (GO)/Kyoto Encyclopedia of Genes and Genomes (KEGG) enrichment analysis is the most universally used and comprehensive functional enrichment method in current medical research. We used the "enrichplot" and "clusterProfiler" packages in R to complete the analysis of the extracted modular signature genes in WGCNA to identify biological processes (BP), cellular components (CC), molecular functions (MF) and pathways. *P*<0.05 was significant.

### Differential Expression Analysis and identification of prognosis-related genes of EAOC

The gene expression matrix of EAOC (GSE73614) was analysed for differential expression using the "limma" package. Log2|Fold Change (FC)| > 1 and FDR< 0.05 were regarded as significant. The module genes obtained from WGCNA were intersected with the DEGs of EAOC. Combined with the clinical information from the GSE73614 dataset, the intersected genes were used for univariate Cox proportional hazard regression (PHR) analysis to screen for genes associated with the prognosis of EAOC at *P*<0.01.

### Cell culture and transfection

Human normal ovarian epithelial cell line IOSE80 was purchased from iCell Bioscience Inc (Shanghai, China), human ovarian clear cell carcinoma cell line ES-2 was purchased from Procell Life Science&Technology (Wuhan,China) and human ovarian endometrioid carcinoma cell line TOV-112D was purchased from FuHeng Biology (Shanghai, China). Cells were cultured in DMEM (Gibco) supplemented with 10% fetal bovine serum (FBS, Gibco), 1% penicillin/streptomycin (Gibco), and incubated at 37°C with 5% CO_2_. All cell lines were subjected to cell line identification and routine mycoplasma detection using short tandem repeat spectroscopy. Logarithmic growth phase cells were selected for functional experiments. The cells were transfected with si-NC, si-ADAMTS19 (RiboBio, stB002365C) and oe-NC, oe-TUBB (RiboBio, stB002564B) using Lipofectamine 3000 reagent (Invitrogen) according to the manufacturer's instructions to achieve knockdown of ADAMTS19 and overexpression of TUBB.

### RNA Extraction and RT-qPCR

Total RNA was extracted from the cells with Trizol reagent (Vazyme) and all samples were reverse transcribed to cDNA, and RT-qPCR was performed by 7500 fluorescence quantitative PCR reactor (ABI, USA) using β-actin as an internal reference gene. The mRNA expression in the samples was calculated using the formula Folds=2^-∆∆CT^. The primer sequences were as follows:

ADAMTS19: F: 5' ACACTATCCCATCAGACCCTC 3'

R: 5' TCTTCCTTTCTCCTCCTCCA 3';

TUBB: F: 5' ATTTCTTTATGCCTGGCTTTG 3'

R: 5' GACCTGCTGGGTGAGTTCC 3';

β-actin: F: 5' CGTGGACATCCGCAAAG 3'

R: 5' AAGGTGGACAGCGAGGC 3'

### Immunohistochemistry (IHC) staining

According to Cohen (2013), the ideal statistical test power and effect size need to be higher than 0.8. Using this as a criterion, the total sample size required in a one-way two-level between-groups design was estimated using the GPower software (http://www.gpower.hhu.de/) to be 42. We collected paraffin embedded specimens of 22 cases of EAOC (20 cases of ovarian clear cell carcinoma, 2 cases of ovarian endometrioid carcinoma) and 20 cases of normal ovarian tissue from the pathology department of our hospital for immunohistochemical study. None of the patients had been treated by chemotherapy or radiation therapy before resection of the primary ovarian cancer and ineligible patients will be excluded. This study was approved by the Ethics Committee of the Second Affiliated Hospital of Kunming Medical University. After antigen retrieval, samples were blocked with 10% BSA and incubated with ADAMTS19/TUBB primary antibody (Proteintech) in a wet box at 4°C overnight, followed by ready-to-use immunohistochemical amplification for 30 min and HRP (mouse, rabbit) reagents for 1 h. The colour was developed by adding 50 μL of DAB colour developing reagent dropwise, followed by counterstaining with haematoxylin. The percentage of microscopically positive cells and staining intensity were scored by semi-quantitative interpretation. 5 fields of view were observed on each section. The percentage of positive cells is graded on a scale of 0 to 4, where <5% is 0, 5%-25% is 1, 26%-50% is 2, 51%-75% is 3, and 76%-100% is 4. The degree of positive staining is also graded on a scale of 0 to 3, where colourless is 0, pale yellow is 1, tan is 2, and brown is 3. The positive grade is obtained by multiplying the two scores.

### Cell Counting Kit-8

Cell viability was determined using Cell Counting Kit-8 (DOJINDO). 2000 cells per well were cultured in 96-well plates with 3 replicate wells. Each well was spiked with 10 μl of CCK-8 reagent. After incubation for 1 h, the absorbance at 450 nm was detected by a microplate reader (ELX800).

### Transwell invasion assay

The upper chamber surface of the membrane at the bottom of the Transwell was coated with a 50 mg/L Matrigel 1:8 (250ul) dilution, the basement membrane was hydrated, a cell suspension of 3×10^4^ cells/200µl was taken and added to the Transwell, and the lower chamber of the 24-well plate was added with 500 µl of complete medium containing FBS, and the cells were cultured in an incubator at 37°C for 24 h. After that 4% paraformaldehyde was added for fixation, stained with 0.1% crystal violet staining solution, and photographed by microscope.

### Statistical analysis

Experimental data were expressed as mean ± standard deviation, and each set of experiments was repeated at least three times. Statistical analyses were performed using GraphPad Prism (v9.1.0). Independent samples t-test was used for comparisons between two groups, one-way analysis of variance (ANOVA) was used for comparisons between multiple groups. Differences were considered statistically significant at *P*<0.05.

## Results

### Identification of the EM-associated modules

**Figure 2:**
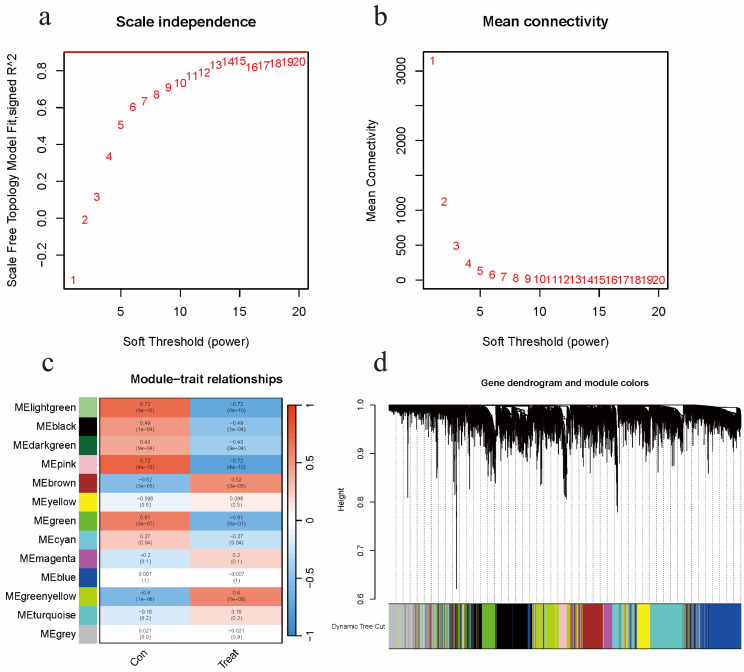
Identification of the EMs-Associated Modules. (a) Analysis of the scale-free fit index for various soft-thresholding powers β. (b) Analysis of the mean connectivity for various soft-thresholding powers. (c) Heatmap of the correlation between module eigengenes and clinical traits of EMs. (d) Dendrogram based on a dissimilarity measure (1-TOM).

A total of 16,077 genes were included after merging the GSE7305 and GSE120103 datasets and removing the batch effect. The WGCNA package was used for mRNA co-expression network analysis, and the optimal β value was determined to be 15 by selecting β. Scale-free network validation was performed for β = 15 (Figures 2a-b). Finally, a total of 13 modules were identified. Modules associated with EMs were evaluated and it was concluded that modules with greater Module Significance (MS) were more associated with disease progression (Figures 2c-d). The ME in the lightgreen and pink modules were clinically more significant for disease progression than any other disease module and were selected for further analysis. The two aforementioned modules included a total of 615 genes.

### Functional annotation and analysis of EMs modular genes

**Figure 3:**
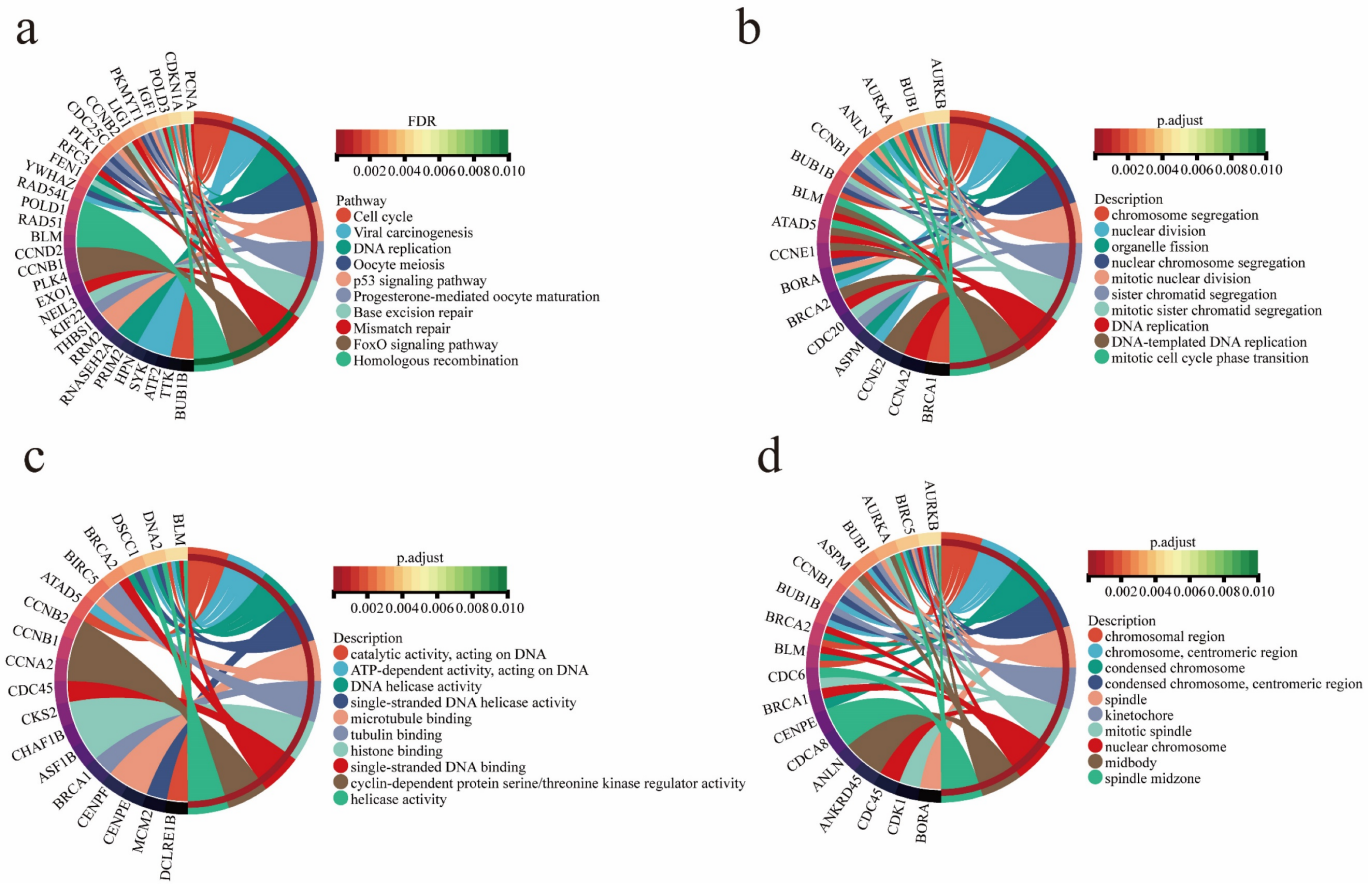
Functional Annotation and Analysis of EMs modular genes. (a) Gene enrichment in tumor-associated pathways. (b) Gene enrichment in biological processes. (c) Gene enrichment in molecular functions. (d) Gene enrichment in cellular components.

Functional annotation, KEGG pathway and GO functional enrichment analyses were performed on two gene modules closely related to EMs. The results showed that these genes were significantly enriched in progesterone-mediated oocyte maturation, oocyte meiosis, viral carcinogenesis, cell cycle, P53 signaling pathway, and other tumor-associated pathways (Figure 3a). The tumor pathways involved in EMs-related genes regulate the development of EAOC to some extent. GO function enrichment analysis showed that the genes were enriched in chromosome segregation, nuclear division, DNA replication and other biological functions (Figure 3b). Furthermore, the analyses revealed that the genes were enriched in molecular functional categories such as single-stranded DNA helicase activity and tubulin binding (Figure 3c), as well as in cellular components such as chromosomal region and mitotic spindle (Figure 3d).

### Identification of DEGs in EAOC

**Figure 4:**
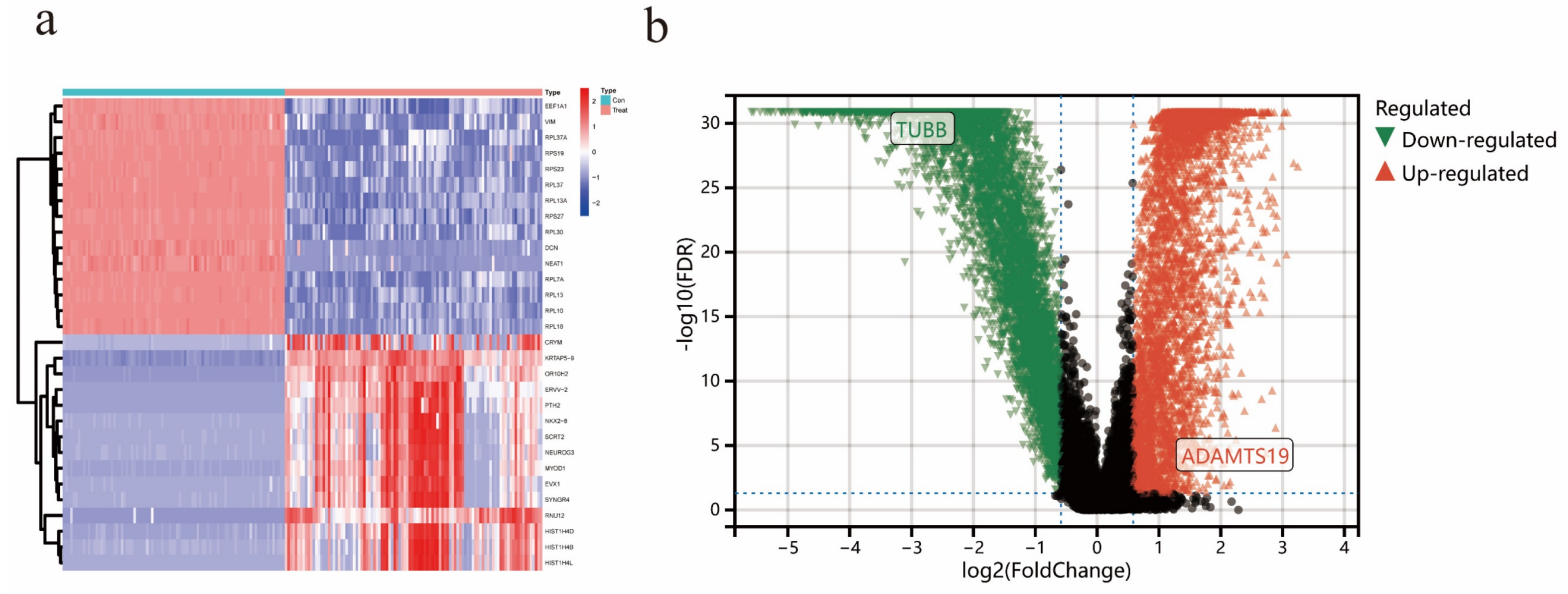
Identification of DEGs in EAOC. (a) Heatmap of the top 15 upregulated and top 15 downregulated genes. (b) Volcano map of DEGs.

In the EAOC dataset GSE73614, a total of 7642 differential genes were identified, including 4034 up-regulated and 3608 down-regulated genes. The heatmap (Figure 4a) showed the top 15 genes that were up-regulated and down-regulated, respectively, and the volcano plot (Figure 4b) showed the expression patterns of DEGs.

### Identification of genes associated with EAOC prognosis

**Figure 5:**
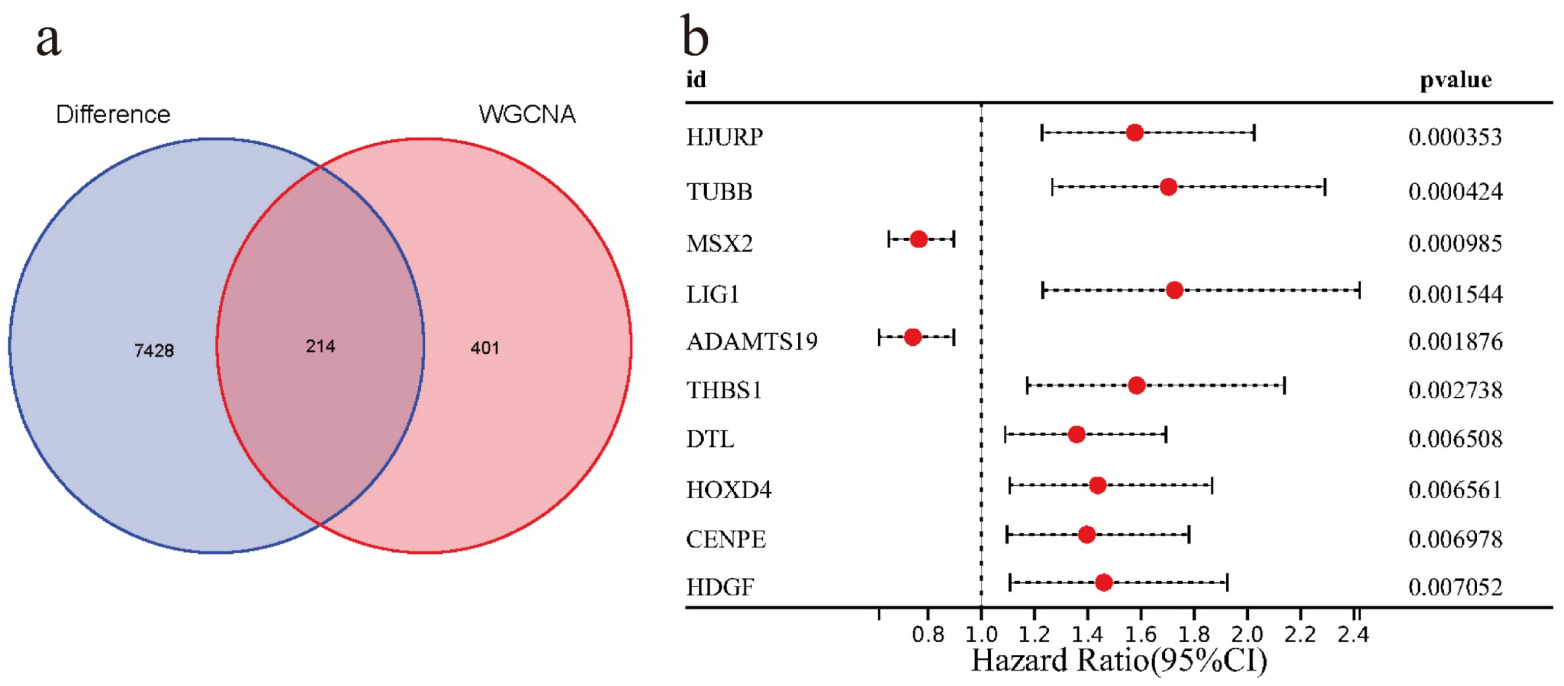
Identification of genes associated with EAOC prognosis. (a) The Venn diagram shows that there are 214 intersecting genes between the modular signature genes of EMs and the differential genes of EAOC. (b) Univariate COX analysis of prognostic factors in patients with EAOC.

In this part, we first intersected 615 EMs module feature genes screened in WGCNA analysis with 7642 EAOC differential genes, and a total of 214 intersection genes were obtained (Figure 5a).Then the intersection genes were analyzed by univariate COX analysis (P<0.01), 10 genes related to EAOC prognosis were identified, which were as follows: HJURP, TUBB, MSX2, LIG1, ADAMTS19, THBS1, DTL, HOXD4, CENPE, HDGF, among which the Hazard Ratios of MSX2 and ADAMTS19 were <1, while the rest were >1 (Figure 5b).

### The expression of ADAMTS19 and TUBB in EAOC

**Figure 6:**
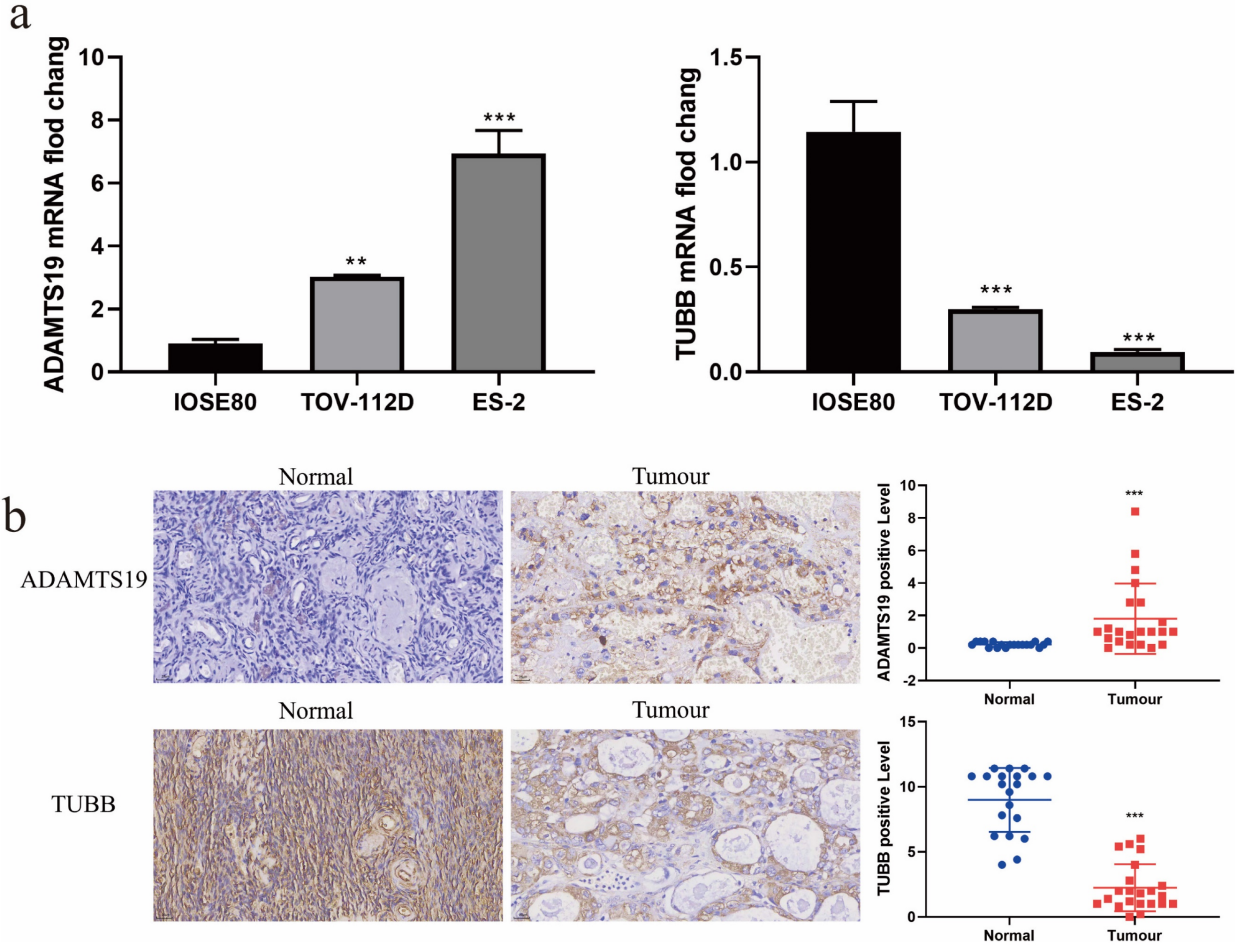
The expression of ADAMTS19 and TUBB in EAOC. (a) The expression levels of ADAMTS19 and TUBB were detected by RT-qPCR in the normal ovarian epithelial cell IOSE80 and the ovarian clear cell carcinoma cell ES-2, and the ovarian endometrioid carcinoma cell TOV-112D. (b) Representative images of ADAMTS19 and TUBB proteins immunohistochemical staining of ovarian tissue sections from patients in the Normal and Tumour groups, scale bar = 25 μm. (**: *P*<0.01, ***: *P*<0.001).

Interestingly, we noticed that among these 10 prognostic-related genes, the multiplicity of difference of TUBB was the largest (|log FC|=3.34), and we learned from our review of the literature that no studies on ADAMTS19 in EAOC have been reported so far. Therefore, we chose ADAMTS19 and TUBB for further analysis. We examined the mRNA and protein expression of ADAMTS19 and TUBB in normal ovarian epithelial IOSE80 cell and ovarian clear cell carcinoma ES-2 cell and ovarian endometrioid carcinoma TOV-112D cell by RT-qPCR and immunohistochemical staining. The results showed that ADAMTS19 expression was up-regulated and TUBB expression was down-regulated in ES-2 and TOV-112D cells compared with IOSE80 cells (Figure 6a-b).

### Cellular assays to evaluate the functions of ADAMTS19 and TUBB in EAOC

**Figure 7:**
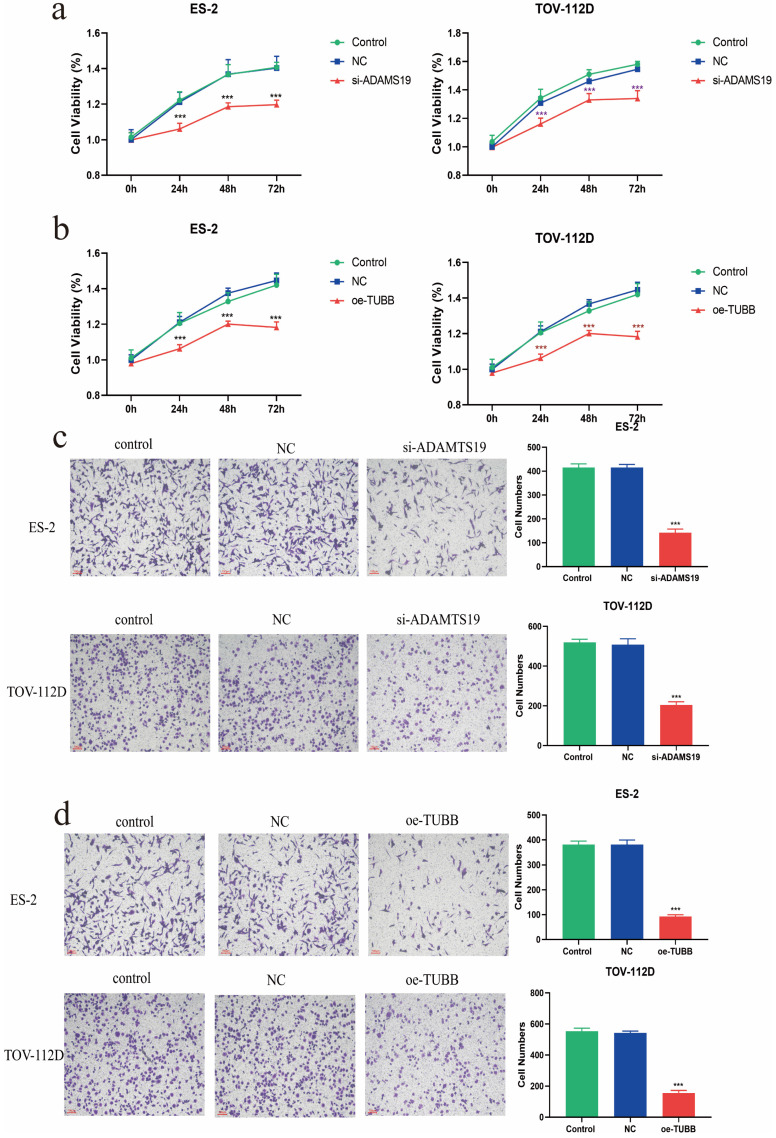
Cellular assays to evaluate the functions of ADAMTS19 and TUBB in EAOC. (a) CCK8 assay detected the cell viability of ES-2 and TOV-112D cells in Control, NC, and si-ADAMTS19 groups. (b) CCK8 assay detected the cell viability of ES-2 and TOV-112D cells in Control, NC, and oe-TUBB groups. (c) Transwell assay detected the effects of ADAMTS19 on the invasive ability of ES-2 and TOV-112D cells. (d) Transwell assay detected the effects of TUBB on the invasive ability of ES-2 and TOV-112D cells. (***: *P*<0.001).

The viability of ES-2 and TOV-112D cells in both si-ADAMTS19 and oe-TUBB groups was significantly decreased at 24h, 48h, and 72h based on CCK8 assays (Figure 7a-b). The Transwell assay demonstrated a significant reduction in the invasive ability of ES-2 and TOV-112D cells in the si-ADAMTS19 and oe-TUBB groups compared to the control group (Figure 7c-d).

## Discussion

**Figure 8:**
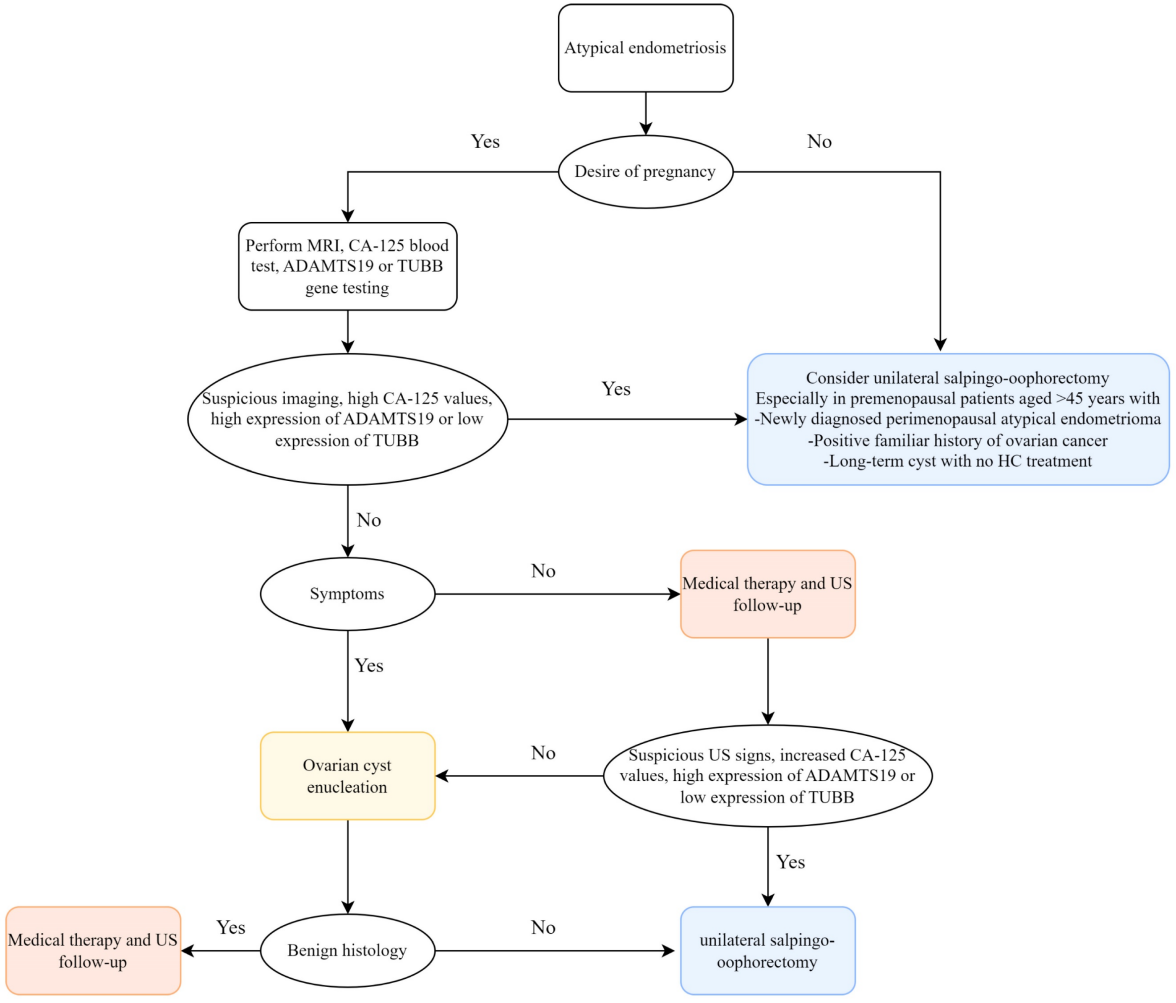
Flowchart for management of ovarian atypical endometriosis. Abbreviations: MRI, magnetic resonance imaging; HC, hormonal contraceptive; US, ultrasound.

Globally, EMs is a common and complex estrogen-dependent inflammatory disease in gynecology^18^, with pelvic pain and low fertility being its most prominent manifestations, having a serious adverse impact on the lives of women of fertile age and placing an economic burden on health systems^19^. EAOC, which includes clear cell carcinoma and endometrioid carcinoma of the ovary, is a subgroup of epithelial ovarian cancers (EOCs)^20^. Sampson first suggested a potential correlation between EMs and malignant transformation of ovarian cancers in the 1920s^21^, and as time passed, more and more literatures have reported that EMs is a risk factor for EAOC, which is clinically, genomically and immunologically caused by EMs lesions^14^, ^22^-^25^. Cases of malignant transformation in perimenopausal women with bladder endometriosis, bowel endometriosis, ovarian endometriosis, thoracic and neurological endometriosis were reported by L. Alio *et al.*^26^. Several studies have indicated atypical endometriosis (AE)—i.e., the histological finding of cytologic atypia and architectural atypia or hyperplasia—as the direct precursor to these specific tumor histotypes: AE is present in 12-35% of ovarian endometriosis cases, and approximately 60-80% of EAOC occurs with AE^27^. The specific oncogenic mechanisms of progression from AE to EAOC are oxidative stress-induced DNA damage, ARID1A and PIK3CA mutations, and microenvironmental factors such as inflammation and tumour immunity^15^, ^16^, ^28^, ^29^. An early medical and/or surgical treatment may reduce disease progression with an immediate improvement in quality of life and fertility. Taking into consideration the existing literatures and our study, we recommend early prevention of disease progression to avoid EMs from developing into EAOC. However, up to now, the molecular mechanisms underlying the complex interactions between EMs and EAOC are not completely understood, and exploring the relationship between EMs and EAOC is important for patient treatment and prognosis. We identified shared genes and common features of EMs and EAOC through bioinformatics approaches and validated them at the cellular level and in patient tissues, with a view to monitoring carcinogenesis at the EMs stage and better treatment and timely prevention of EAOC. Based on the study by Gabriele *et al.*^27^, we also propose a pragmatical clinical flowchart to optimize the available therapeutic options, favoring patient quality of life (Figure 8).

In this study, we screened EMs-related modules from 16077 genes by WGCNA analysis, and we also obtained 7642 differential genes of EAOC. Then, the intersection of EMs-related genes and the differential genes of EAOC was taken to obtain the shared genes for univariate COX analysis (*P*<0.01). Ultimately, 10 genes related to the prognosis of EAOC were screened: HJURP, TUBB, MSX2, LIG1, ADAMTS19, THBS1, DTL, HOXD4, CENPE, HDGF. Among these 10 genes, we selected ADAMTS19 and TUBB as target genes to further analyse their value in EAOC.

ADAMTS19 is capable of encoding a member of the ADAMTS family of proteins. Jiang *et al.* reported that ADAMTS19 expression was decreased in gastric cancer, that it inhibited gastric cancer cell migration and invasion by targeting S100A16 through the NF-κB pathway, and that ADAMTS19 was a potential metastatic and survival biomarker for gastric cancer^30^. Furthermore, it has been shown that hypermethylation of the ADAMTS19 gene is prevalent in gastrointestinal cancers and that epigenetic inactivation of ADAMTS19 promotes metastasis and spread of colorectal cancer^31^. In our study, by RT-qPCR and immunohistochemistry, we confirmed that the expression of ADAMTS19 was upregulated in both EAOC cells and tissues compared with normal ovarian cells and tissues, and more importantly, we found that ADAMTS19 was able to promote the proliferation and invasion of EAOC cells by CCK8 and Transwell assays.

TUBB (Tubulin β class I) encodes β-microtubulin. TUBB binds to α-tubulin to form a dimer, which, as a component of eukaryotic microtubules, is involved in cell division, intracellular signaling and transport^32^, ^33^. Previous studies have shown that TUBB plays multiple pathological roles. It was a highly expressed isoform of β-microtubule protein in most epithelial tumour cells and was positively associated with poorer prognosis, metastasis, and resistance to microtubule-targeting agents in lung adenocarcinoma^34^-^36^. For example, in high-grade serous ovarian cancer, carboplatin induced upregulation of TUBB expression, which affected acquired resistance and cross-resistance in patients^37^. Furthermore, TUBB was highly expressed in cutaneous melanoma and positively correlated with the malignant behaviour of the tumour such as epithelial-mesenchymal transition (EMT), invasion and metastasis^38^. This study confirms the down-regulation of TUBB expression in EAOC at the cellular and tissue levels through RT-qPCR and immunohistochemistry. Functional experiments revealed that TUBB effectively suppressed the proliferation and invasion of EAOC cells.

To the best of our knowledge, no studies of ADAMTS19 and TUBB in EAOC have been reported, and our study provides the first preliminary insight into the biological functions of ADAMTS19 and TUBB in EAOC. However, this study has some limitations. In this retrospective study, we focused only on microarray expression cohorts with a small sample size, which may lead to selection bias. Some significant genes may have been missed during the multi-step screening process, which limited the identification of prognosis-related genes. Besides, in performing functional exploration of ADAMTS19 and TUBB, we just simply performed CCK8 and Transwell assays. Subsequent research should be conducted on larger sample sizes and add functional experiments such as clone formation, cell cycle, and apoptosis to validate at the cell, tissue, and animal levels. Moreover, additional research is required to examine the precise mechanisms through which ADAMTS19 and TUBB impact the prognosis of EAOC. More importantly, we need to discuss in depth a potential diagnostic/therapeutic flow-chart list.

Biomarkers have a wide range of applications in clinical medicine. At present, more and more potential biomarkers are being discovered, and their study as new targets is a hotspot for new drug development in various therapeutic areas, especially in the field of tumour therapy. There is growing evidence that the clinical benefits of genetic testing outweigh the costs, despite its high cost^39^, ^40^. With rapid advances in gene microarray technology, researchers can measure the expression levels of many thousands of genes in a short period, which aids in the deeper understanding of the pathogenesis of diseases at the genetic level^41^. Genetic biomarkers will become more widely used in the future and would be regularly employable.

## Conclusions

In conclusion, we identified 10 genes related to the prognosis of EAOC based on the cohort of GEO database using bioinformatics methods. We also validated the expression levels of ADAMTS19 and TUBB in EAOC and their respective biological functions. This study has enhanced our comprehension of the correlation between EAOC and EMs. These gene markers show potential as valuable prognostic biomarkers and as possible therapeutic targets for EAOC patients.

## Figures and Tables

**Table 1:** Detailed information of GEO and GTEx datasets containing the EMs and EAOC patients.

ID	Type of database	Platform	Samples	Disease	Group
1	GEO-GSE7305	GPL570	10 patients and 10 controls	EMs	WGCNA analysis
2	GEO-GSE120103	GPL6480	18 patients and 18 controls	EMs	WGCNA analysis
3	GEO-GSE73614	GPL6480	37 OCCC and 66 OEC	EAOC	DEGs analysis
4	GTEx	-	88 normal ovarian tissues	-	DEGs analysis

GEO: Gene Expression Omnibus database; GTEx: Genotype-Tissue Expression database; OCCC: Ovarian clear cell carcinomas; OEC: Ovarian endometrioid carcinomas; EMs: Endometriosis; EAOC: Endometriosis-associated ovarian cancer; WGCNA: Weighted gene co-expression network analysis; DEGs: Differentially expressed genes

## Abbreviations

EMs: Endometriosis
EAOC: Endometriosis-associated ovarian cancer
OCCC: Ovarian clear cell carcinomas
OEC: Ovarian endometrioid carcinomas WGCNA: The weighted gene co-expression network analysis
KEGG: Kyoto Encyclopedia of Genes and Genomes
GO: Gene Ontology
GEO: Gene Expression Omnibus
ADAMTS19: ADAM metallopeptidase with thrombospondin type 1 motif 19
TUBB: Tubulin beta class I
RT-qPCR: Quantitative reverse transcription polymerase chain reaction
CCK8: Cell counting kit 8
DEGs: Differentially expressed genes
TNF-α: Tumor Necrosis Factor α
IL-8: Interleukin-8
VEGF: Vascular endothelial growth factor
PTEN: Phosphatase and tensin homolog
HNF-1β: HNF1 homeobox B
NCBI: The National Center for Biotechnology Information
GTEx: Genotype-Tissue Expression
TOM: Topological overlap matrix
BP: Biological processes
CC: Cellular components
MF: Molecular functions
FC: Fold change
FDR: False discovery rate
PHR: Proportional hazard regression
FBS: Fetal bovine serum
IHC: Immunohistochemistry
HRP: Horse radish peroxidase
DBA: Diaminobenzidine
ANOVA: Analysis of variance
MS: Module significance
HR: Hazard ratios
OS: Overall survival
EOC: Epithelial ovarian cancers
AE: atypical endometriosis
